# Braithwaite’s Retrospect

**Published:** 1866-05

**Authors:** 


Braithwaite's Retrospect.—In the next July number of the "*Retrospect*," an American Appendix will be added, entitled "*Half-Yearly Digest of the Medical Sciences;*" comprising a practical summary of every discovery and improvement in medicine and surgery in the United States, during the previous six months.

The subscription to "*Braithwaite*," including the American "*Digest*," $3.00; when not included, $2.50; the "*Digest*" separately, 80 cents a year. Single numbers, 50 cents.

